# Mother–Father Distress, Accommodation, and Child Eating Disorder Behaviors: A Dyadic Perspective

**DOI:** 10.1111/famp.70070

**Published:** 2025-09-04

**Authors:** Alexandra Bédard, Marilou Côté, Dominique Meilleur, Giuseppina Di Meglio, Nathalie Gingras, Caroline Pesant, Danielle Taddeo, Richard Bélanger, Isabelle Thibault, Pierre‐Olivier Nadeau, Holly Agostino, Chantal Stheneur, Jean‐Yves Frappier, Catherine Bégin

**Affiliations:** ^1^ Centre Nutrition, Santé et Société (NUTRISS), Institut Sur la Nutrition et les Aliments Fonctionnels (INAF) Université Laval Québec Québec Canada; ^2^ Département des Fondements et Pratiques en Éducation, Faculté des Sciences de l'Éducation Université Laval Québec Québec Canada; ^3^ Département de Psychologie Université de Montréal Montréal Québec Canada; ^4^ Hôpital de Montréal Pour Enfants, Centre Universitaire de Santé McGill Montréal Québec Canada; ^5^ Centre de Pédopsychiatrie, Centre Intégré Universitaire de Santé et de Services Sociaux de la Capitale‐Nationale Québec Québec Canada; ^6^ Département de Psychiatrie et de Neurosciences, Faculté de Médecine Université Laval Québec Québec Canada; ^7^ Hôpital Fleurimont, Centre Intégré Universitaire de Santé et de Services Sociaux de l'Estrie ‐ Centre Hospitalier Universitaire de Sherbrooke Sherbrooke Québec Canada; ^8^ Centre Hospitalier Universitaire Sainte‐Justine Montréal Québec Canada; ^9^ Département de Pédiatrie, Faculté de Médecine Université Laval Québec Québec Canada; ^10^ Centre Hospitalier Universitaire de Québec Québec Québec Canada; ^11^ Département de Psychoéducation Université de Sherbrooke Sherbrooke Québec Canada; ^12^ Département de Pédiatrie, Faculté de Médecine Université de Montréal Montréal Québec Canada; ^13^ École de Psychologie, Université Laval Québec Québec Canada

**Keywords:** accommodation, anorexia nervosa, anorexic behaviors, dyadic analysis, parents, psychological distress

## Abstract

Parental psychological distress and accommodating and enabling behaviors may represent maintaining factors of anorexia nervosa (AN). However, very few studies included both parents; their interdependence is unknown. Using a dyadic approach, this study aimed to examine the relationship between parental psychological distress and accommodation at the admission of their child to specialized eating disorder programs, and their observation of their child's eating disordered behaviors 1 year later. Ninety‐one dyads of mixed‐gender couples of parents of children and adolescents diagnosed with AN (*M*_age_ = 14.5 ± 1.5 years) were recruited from one of the five University Health Centers across the province of Québec, Canada. At admission, parents completed the Psychological Distress Index and the Accommodation and Enabling Scale for Eating Disorders. Furthermore, parents reported their child's anorexic behaviors 12 months later using the Anorexic Behavior Observation Scale. The dyads were nondistinguishable by gender, suggesting a similar pattern of associations for mothers and fathers. Path analyses guided by the actor–partner interdependence model revealed an indirect effect within each parent; higher parental psychological distress was associated with higher child's eating disordered behaviors at the 12‐month follow‐up through greater parental eating disorder accommodation. A partner effect was also found; when one parent experienced psychological distress, the other parent was more likely to engage in concomitant accommodating behaviors, which, in turn, was associated with a report of more child's eating disordered behaviors by this parent at the 12‐month follow‐up. These findings highlight the importance of a dyadic perspective in exploring parents' emotional states and behaviors toward children with AN.

## 1 Introduction

Anorexia nervosa (AN) is a serious life‐threatening psychiatric condition that predominantly develops in adolescence (van Eeden et al. 2021). Without early treatment, AN can be accompanied by severe medical complications (Golden et al. 2003; Meczekalski et al. 2013; Rome et al. 2003), and high morbidity and mortality rates (Arcelus et al. 2011; Fichter et al. 2006; Preti et al. 2011; Sullivan 1995; van Eeden et al. 2021). Despite this, parents report difficulties in acquiring needed specialty treatment (Wilksch 2023), and when acquired, many patients deny the seriousness of their illness and are strongly resistant to treatment (Fassino and Abbate‐Daga 2013; Viglione et al. 2006). This situation may be highly challenging and stressful for parents, who are usually the primary caregivers of the child with AN (Couturier et al. 2020; National Institute for Health and Care Excellence 2017).

Previous research has shown that caregivers of children and adolescents with AN are particularly vulnerable to experiencing psychological distress, with more than one‐third of them reaching a clinically significant level (Anastasiadou et al. 2014; Rhind et al. 2016; Whitney et al. 2007). The results of a survey conducted among 439 parents of children with eating disorders (ED; 81.9% were diagnosed with AN) reported that the rate of parents with clinical levels of distress during their child's illness was four to six times greater than that of parents of recovered children (Wilksch 2023). Evidence‐informed guidelines advise including parents in the treatment and care of children and adolescents (National Institute for Health and Care Excellence 2017). Research supports the effectiveness of family‐based therapy (FBT; Couturier et al. 2020; National Institute for Health and Care Excellence 2017), and parent involvement is a component of other approaches like enhanced cognitive‐behavior therapy (Le Grange et al. 2020). Parents of young patients play a key role as active participants in the therapeutic process, assuming responsibility for their child's nutritional rehabilitation and overall well‐being. Accordingly, emphasis has been placed on providing support to parents as they engage in the challenging tasks they are undertaking (Gorrell and Le Grange 2023). The mechanism of change in FBT often centers around the concept of parental self‐efficacy (Lock et al. 2015; Robinson et al. 2013). This refers to the parents' belief in their ability to effectively manage and address their child's eating disordered behaviors. Despite the importance of parents' self‐efficacy as a change factor, parents generally perceive that they lack skills and resources to fulfill their role as caregivers (Haigh and Treasure 2003; Sepulveda et al. 2008) and, combined with worries about the illness and a high level of caring demands, this can lead to psychological distress (Dimitropoulos et al. 2008; Fox and Whittlesea 2017; Haigh and Treasure 2003; Treasure et al. 2001; Whitney and Eisler 2005). Parental distress can also influence the sense of self‐efficacy among parents by creating an emotional and psychological context that may compromise their perception of their ability to cope with the challenges associated with caring for their child with ED (Gondoli and Silverberg 1997). There is some evidence that parents' emotional responses can impact the prognosis and treatment of AN (Anastasiadou et al. 2014; Zabala et al. 2009), although some studies have not observed this link (Matthews et al. 2023).

In response to diminished emotional resources, parents can be more inclined to tolerate or accommodate AN behaviors, becoming organized around ED rules (accommodating to the illness) and ignoring or covering up the negative consequences of the behaviors (enabling the illness; Fox and Whittlesea 2017; Treasure and Schmidt 2013). Accommodating and enabling behaviors are highlighted as key maintaining mechanisms in the cognitive‐interpersonal model of AN (Janet et al. 2020; Schmidt and Treasure 2006; Treasure and Schmidt 2013). This model postulates that caregivers experiencing high levels of anxiety and psychological distress are more inclined to accommodate and enable their loved one's AN behaviors in an effort to reduce conflict and distress (Treasure and Schmidt 2013). By allowing the person with AN to engage in behaviors that do not promote recovery, the illness is maintained. Initial research has supported the cognitive‐interpersonal model, indicating that high levels of parental accommodation relate to poorer treatment outcomes in patients with AN (Anderson et al. 2021; Monteleone et al. 2022; Salerno et al. 2016; Wagner et al. 2020).

Most of the existing research has focused on mothers and little is known about the impact of fathers' emotional state and behaviors on the recovery of a child with AN (Anastasiadou et al. 2014; Matthews et al. 2023). Rhind et al. (2016) did study both mothers and fathers and found that the higher the level of parental distress is, the more the parent is inclined to accommodate, irrespective of the parent's gender. In addition, findings from Salerno et al. (2016) suggest that not only the mother's accommodation would impact the child's recovery, but also the accommodating behaviors of the father. More precisely, their findings suggest a dose‐dependent relation between family accommodation and patients' symptom severity over 12 months. Patients' symptom trajectory was worse when both mother and father were high in accommodating and enabling behaviors, and better when neither parent was accommodating and enabling. When one parent engaged in accommodation while the other did not, patients exhibited intermediate symptom outcomes, irrespective of which parent was engaging in accommodation. Their results also demonstrated that high accommodating behaviors among fathers had an independent effect from the level of mothers in perpetuating the child's AN symptoms (Salerno et al. 2016). Although only a few studies have been carried out so far, these results suggest the need to consider not only mothers but also fathers in our research efforts, even though fathers are often considered secondary caregivers.

There has been a scarcity of studies incorporating perspectives from both mothers and fathers. More importantly, despite the influence of other family members on each other's psychological health and behaviors within a shared context, the mutual reliance between mothers and fathers has been disregarded so far. Yet, the cognitive‐interpersonal theoretical model proposes that caregivers may accommodate AN behaviors to alleviate their own stress and negative affect, but also to reduce the stress and negative affect of other family members (Treasure and Schmidt 2013), thus supporting the relevance of examining the interdependence between parents. When interdependency is expected, the traditional regression methods are not advisable due to their assumptions of independent observations (Kenny and Judd 1986). In that context, the actor–partner interdependence model (APIM) becomes particularly relevant, as it accounts for the interdependence of parents by examining how variables interact within parents (i.e., actor effects), but also between parents (i.e., partner effects; Cook and Kenny 2005). Therefore, to shed some light on the interconnectedness between parents, the present study proposes using the APIM to examine the relationship between parental psychological distress and accommodation at the admission of their child to specialized eating disorder programs, and their observation of their child's eating disordered behaviors 1 year later, and to determine whether these effects are different according to the parent's gender (i.e., mothers vs. fathers). Since previous studies suggest that the relationships between parental psychological distress and accommodation, as well as between parental accommodation and the child's eating disordered behaviors, are similar in mothers and in fathers (Rhind et al. 2016; Salerno et al. 2016), we hypothesized that, in both parents, a higher psychological distress would be associated with greater accommodating and enabling behaviors, which in turn would be associated with the observation of higher child's eating disordered behaviors 12 months later. We also expected a partner effect. When one partner experienced psychological distress, the other partner would accommodate AN behaviors to alleviate the partner's negative affect, which in turn would also be associated with a heightened perception of the child's eating disordered behaviors 12 months later, regardless of the parent's gender.

## 2 Method

### 2.1 Participants

This prospective study recruited children and adolescents attending the specialized ED programs (inpatient or outpatient) at five University Health Centers across the province of Québec, Canada. Eligible participants were children and adolescents diagnosed with AN, atypical AN (meeting the same criteria as AN except that despite significant weight loss, the individual's weight remained within or above the normal range), or avoidant/restrictive food intake disorder (ARFID) according to DSM‐5 criteria (American Psychiatric Association 2013). Diagnosis was confirmed by clinicians with expertise in pediatric ED. Recruitment was carried out from May 2016 to June 2020.

Two primary caregivers were also invited to participate. Inclusion criteria for caregivers were: (a) being one of the young person's primary caregivers, and (b) providing written informed consent. Although other close caregivers were encouraged to participate, the present analyses focused solely on parental couples (biological or adoptive mother and father) who were still in a romantic, cohabiting relationship. Only parents of youth diagnosed with AN were included. Youth with ARFID were excluded. Due to the dyadic nature of the analyses, both partners also needed to have completed at least one of the relevant questionnaires. Same‐gender couples were initially included, but missing data for at least one partner led to their exclusion from the final sample, which therefore consists only of mixed‐gender couples.

The study was conducted in accordance with the Declaration of Helsinki and approved by the Ethics Committee of the coordinating center (CHU de Québec‐Université Laval; protocol: MP‐20‐2015‐2323; date of approval: October 30, 2015). Site‐specific ethics approval was also granted at all participating sites.

### 2.2 Procedure

At the admission of their child to ED services, both parents were asked to complete online questionnaires about their sociodemographic characteristics, their psychological distress, and their accommodating and enabling behaviors. At admission, the attending ED clinician also reported the patient's medical characteristics. Furthermore, both parents had to report their child's eating disordered behaviors 12 months later.

Treatment involved interdisciplinary care, encompassing family and individual therapy sessions, nutrition counseling, meal support, psychiatric and medical monitoring, and additional therapeutic interventions (e.g., art therapy).

### 2.3 Measures

#### 2.3.1 Sociodemographic and Clinical Characteristics

Parents were asked to report their sociodemographic characteristics (e.g., gender, age, ethnicity, educational level) and those of their child with AN. They were also asked to report the number of hours they spent with their child by answering the following questions: “On average, how many hours per day do you spend with your child during a typical weekday?” and “On average, how many hours per day do you spend with your child during a typical weekend day?”. AN patient's medical characteristics (e.g., typical/atypical AN diagnosis, inpatient/outpatient treatment setting) were reported by the medical team.

In addition, the severity of the child's ED behaviors and symptoms was assessed upon admission by the attending ED clinician using the Eating Disorder Symptom Severity Scale (EDS3; Henderson et al. 2010). This instrument assesses the severity of the ED according to five general domains: ED symptoms and behaviors, ED urges, ED cognitions, ED anxiety, and treatment progress. In the present study, the total score was used. Higher scores indicate more severe symptoms. All attending ED clinicians participated in a 90 min training session to ensure that the EDS3 was administered accurately and consistently across clinicians. In this study, internal consistency for the total score was 0.82.

#### 2.3.2 Psychological Distress

The Psychological Distress Index—14 items (IDPESQ‐14) developed by Préville (1992) is a French questionnaire based on Ilfeld's Psychiatric Symptom Index questionnaire (Ilfeld 1976). This questionnaire uses a 4‐point Likert scale (1 = *never*, 2 = *occasionally*, 3 = *somewhat often*, 4 = *very often*) to detect symptoms of anxiety, depression, irritability, as well as cognitive problems. A higher total score indicates a higher level of psychological distress. A validation study revealed adequate internal consistency, with a Cronbach's α of 0.89 for the total 14‐item scale, and good concurrent validity, with significant associations with four other measures of respondents' health status: (a) consultation with a health professional for a mental health problem in the previous 12 months, (b) hospitalization for a mental health problem in the previous 12 months, (c) presence of suicidal ideation or attempt in the previous 12 months, and (d) use of certain psychoactive drugs. In this study, internal consistency for the total scale was 0.90 for mothers and 0.92 for fathers.

#### 2.3.3 Eating Disorder Accommodation

The Accommodation and Enabling Scale for Eating Disorders (AESED) developed by Sepulveda et al. (2009) is a 33‐item questionnaire designed to measure accommodating and enabling behaviors by caregivers of people with ED. Each item is rated on a 5‐point Likert scale (0 = *never*, 1 = *rarely*, 2 = *sometimes*, 3 = *often*, 4 = *nearly always*). This instrument is composed of five themes, namely *avoidance and modifying family routine* (i.e., the extent to which caregivers change their typical activities and behaviors because of their relative's disorder), *reassurance seeking* (i.e., the extent to which caregivers engage in repeated conversations focused on body image, eating, and negative thoughts and feelings), *meal ritual* (i.e., the accommodation of food storage, cooking, and cleaning rules), *control of the family* (i.e., the extent to which their loved one with ED controls the choice of food and cooking practice), and *turning a blind eye* (i.e., the extent to which caregivers ignore signs of ED behaviors). A total score, including all items of the questionnaire, was calculated. A higher score indicated a higher accommodation to ED symptoms. The AESED has shown high internal consistency, with a Cronbach α of 0.92 for the total scale (Sepulveda et al. 2009). In the present study, internal consistency for the total scale was 0.93 for mothers and 0.94 for fathers.

#### 2.3.4 Eating Disordered Behaviors

The Anorectic Behavior Observation Scale (ABOS; Vandereycken 1992) is a 30‐item questionnaire used to evaluate the eating disordered behaviors of patients. It is based on the caregiver's observations made during the last month at home. Each item is rated with three response options (0 = *no*, 1 =? *(I don't know)*, 2 = *yes*). This questionnaire is composed of three domains: (a) eating behaviors, concern with weight and food, denial of problems, (b) bulimic behavior, and (c) hyperactivity. The higher the total score, the greater the patient's eating disordered pathology. Moderate‐to‐high correlations have been previously found between parents' observation of eating disordered behaviors reported by this questionnaire and judgments made by the clinician (Vandereycken 1992). In addition, strong correlation coefficients were observed in the present study at 12 months between the ABOS score and the severity of the child's ED behaviors and symptoms as assessed by the child's attending ED clinician using the EDS3 questionnaire for both mothers (*r* = 0.68, *p* = 0.0002) and fathers (*r* = 0.54, *p* = 0.01). The reliability and validity of the ABOS have been previously supported (Vandereycken 1992). In this study, internal consistency for the total scale was 0.80 for mothers and 0.90 for fathers.

### 2.4 Statistical Analysis

Differences between mothers and fathers were assessed using paired Student's *t*‐tests for continuous variables and chi‐squared tests (five or more participants per cell) or Fisher's exact tests (at least one cell with fewer than five participants) for categorical variables. Pearson's correlations were also run to examine the relationships between psychological distress at baseline, accommodation at baseline, and the child's eating disordered behaviors, as observed by parents, at the 12‐month follow‐up.

A path analysis was conducted to test the hypothesized APIM model using the software M*plus* version 7.0 (Muthén and Muthén 2012). An APIM framework was used because it allows taking into account the interdependence between two members of a dyad (i.e., nested data; Kenny et al. 2006; herein mixed‐gender couples of parents of a child with AN). In the current model, psychological distress at baseline was the independent variable, ED accommodation at baseline was the mediator, and parents' observation of their child's eating disordered behaviors at 12‐month follow‐up was the dependent variable. Also, the tested model included the severity of the child's ED behaviors and symptoms at admission, as reported by the child's attending ED clinician (EDS3 total score), as a covariate. Student's *t*‐tests were used to explore whether differences existed between parents of children diagnosed with typical AN and those with children diagnosed with atypical AN for any variable included in the model. As no differences were observed, the analysis was conducted on the entire group. All actor and partner effects were estimated simultaneously in the model, and no paths were removed even if nonsignificant to control for their associations. Models were tested with maximum likelihood estimation using robust standard errors (MLR; Muthén and Muthén 2012), and bootstrap procedures were used to test whether the indirect effects were significant. To do so, 5000 random samples were drawn with replacement from the original sample to construct bias‐corrected confidence intervals (Edwards and Lambert 2007).

An omnibus chi‐squared difference test was run to verify whether parents were distinguishable by their gender, based on Kenny et al. (2006) recommendations. This test compared the chi‐square when parameters (i.e., variances, intra‐ and interpersonal covariances) were allowed to vary freely across genders to the chi‐square when parameters were constrained to be equal for mothers and fathers. A significant *p* value would indicate distinguishable dyads by gender (i.e., the pattern of variances and covariances would differ significantly between genders), and then it would be appropriate to treat mothers and fathers distinctively in the APIM. Conversely, a nonsignificant chi‐square would indicate indistinguishable dyads by gender (i.e., the pattern of variances and covariances would not be considered different for both genders); then it would be indicated to constrain parameters to be equal across genders. In this scenario, mothers and fathers would be treated as interchangeable.

In the current sample, there were virtually no missing data at baseline for both mothers and fathers across all variables studied, with rates ranging from 0% to 2%. However, parents' observation of their child's eating disordered behaviors (ABOS) assessed at the 12‐month follow‐up was missing for 30.8% of mothers and 42.9% of fathers. To evaluate whether missingness was related to baseline variables, we compared dyads with and without missing data at follow‐up on relevant parental sociodemographic characteristics (age, ethnicity, educational level) and on child medical variables at intake (gender, age, ethnicity, diagnosis [typical vs. atypical AN], setting at admission [inpatient vs. outpatient], weight loss, duration of ED). Student's *t*‐tests were used for continuous variables and chi‐squared tests (five or more participants per cell) or Fisher's exact tests (at least one cell with fewer than five participants) for categorical variables. No difference was observed for any variables studied. Accordingly, missing data were handled using Full Information Maximum Likelihood (FIML). This method uses maximum likelihood to estimate model parameters using all available data (Marcoulides and Schumacker 1996). Sensitivity analysis revealed similar results using listwise or FIML methods, reflecting the robustness of our findings and confirming the relevance of using FIML to optimize the utilization of data collected from parents.

To test whether the model fit the data, several fit indices were used: the chi‐squared statistic, the comparative fit index (CFI), the Tucker–Lewis indices (TLI), and the root mean square error of approximation (RMSEA). The threshold values that were expected for a good fit were a nonstatistically significant chi‐squared value, a value of 0.90 or higher for the CFI and the TLI, and a value below 0.08 for the RMSEA (Hu and Bentler 1999). All variables were z‐standardized prior to the analysis using the mean and the standard deviation computed across mothers and fathers (i.e., using the entire sample).

Student's *t*‐tests were conducted for potential categorical covariates (diagnostic category [typical vs. atypical AN], treatment setting at admission [outpatients vs. inpatients]), and Pearson's correlations for potential continuous covariates (child age, duration of the ED, and weight loss) to examine the relationships between these covariates and the child's eating disordered behaviors reported by parents at the 12‐month follow‐up. If a significant correlation was found between a covariate and the child's eating disordered behaviors reported by parents, the model was adjusted to account for this covariate.

Post hoc power analysis was conducted for the mediation model (*n* = 91, alpha = 0.05, repetition = 5000), for which a power of 0.86 was achieved.

## 3 Results

Of the 288 families who entered the treatment program, 162 in which both caregivers agreed to participate were recruited. Noninclusion at this stage was primarily due to one caregiver declining participation or the research team being unable to reach the other. Among the 162 recruited families, 39 were excluded based on the inclusion/exclusion criteria for the current subanalyses: 26 couples were no longer in a romantic relationship at the time of the study, 11 had a child diagnosed with ARFID or whose diagnosis was not confirmed by the attending clinician, and in two cases, one partner was not the child's biological or adoptive mother or father (i.e., a stepparent). An additional 32 couples were excluded due to missing data from at least one caregiver (i.e., questionnaires not completed despite reminders). Therefore, the study sample included both mothers and fathers of 91 children and adolescents diagnosed with AN. The vast majority (93.4%) of patients (i.e., children and adolescents) were female, with a mean age of 14.5 years (SD = 1.5; range: 10–17 years). Initially, patients were treated in inpatient (*n* = 27; 29.7%) and outpatient (*n* = 64; 70.3%) services, with a diagnosis of typical AN for 76.9% of the patients and atypical AN for 23.1%. Among parents, fathers were slightly older than mothers. Parents were mostly White and highly educated. Mothers reported spending more hours with their child on weekdays and weekend days than fathers. Characteristics of both parents and their child are presented in Table 1.

**TABLE 1:** Characteristics of the parents and their child.

	Mothers (*n* = 91)	Fathers (*n* = 91)	*p*
**Child's characteristics**	*Mean ± SD or n (%)*		
Gender			
Female	85 (93.4)		
Male	6 (6.6)		
Age	14.5 ± 1.5^a^		
Ethnicity			
White	82 (90.1)		
Asian	3 (3.3)		
Latin	3 (3.3)		
African	0		
Other	3 (3.3)		
Diagnostic			
Typical AN	70 (76.9)		
Atypical AN	21 (23.1)		
Setting at admission			
Outpatients	64 (70.3)		
Inpatients	27 (29.7)		
Weight loss before treatment (%)	−16.4 ± 11.5		
Duration of the ED before treatment (days)	358 ± 314		
**Parents' characteristics**	*Mean ± SD or n (%)*	*Mean ± SD or n (%)*	
Age	46.0 ± 4.5	48.2 ± 5.0	< 0.0001
Ethnicity			0.8484
White	84 (92.3)	83 (91.2)	
Asian	2 (2.2)	1 (1.1)	
Latin	3 (3.3)	2 (2.2)	
African	0	1 (1.1)	
Other	2 (2.2)	4 (4.4)	
Educational level			0.3047
High school or less	10 (11.0)	17 (18.7)	
College	17 (18.7)	18 (19.8)	
University	64 (70.3)	56 (61.5)	
Contact with patient (per week day)			0.0005
Less than 1 h	11 (12.1)	33 (36.7)	
Between 1 and 2 h	26 (28.6)	27 (30.0)	
Between 2 and 3 h	29 (31.9)	17 (18.9)	
More than 3 h	25 (27.5)	13 (14.4)	
Contact with patient (per weekend day)			< 0.0001
Less than 1 h	1 (1.1)	4 (4.4)	
Between 1 and 2 h	7 (7.7)	30 (33.3)	
Between 2 and 3 h	19 (20.9)	22 (24.4)	
More than 3 h	64 (70.3)	34 (37.8)	

*Note:* There were 12 missing data points for weight loss before treatment and 11 missing data points for the duration of the ED. One father's data was missing for variables related to the contact with the patient.

Abbreviation: AN, anorexia nervosa.

^a^ Age ranged between 10 and 17 years.

The levels of psychological distress and accommodating behaviors were higher in mothers than in fathers at baseline (Table 2). No differences between parents were noted in the reported level of their child's eating disordered behaviors at the 12‐month follow‐up, that is, mothers and fathers perceived the eating disordered behaviors of their child similarly.

**TABLE 2:** Psychological distress, eating disorder (ED) accommodation, and eating disordered behaviors observation at 12‐month follow‐up in mothers and fathers.

	M ± SD	Paired *t*‐test	1	2	3	4	5	6
1. Mother's psychological distress	29.5 *±* 18.2	−13.40***	—					
2. Father's psychological distress	21.8 *±* 15.6		0.275*	—				
3. Mother's ED accommodation	40.0 *±* 21.5	−4.70***	0.455***	0.176	—			
4. Father's ED accommodation	32.0 *±* 20.8		0.225*	0.379***	0.613***	—		
5. Mother's ED behaviors observation	14.0 *±* 9.2	1.18	0.103	0.120	0.271*	0.247	—	
6. Father's ED behaviors observation	15.4 *±* 11.6		−0.027	0.064	0.238	0.638***	0.515***	—

*Note:* *n* ranged between 63 to 91 for mothers and 52 to 90 for fathers. *p < 0.05; ***p < 0.001.

Abbreviation: ED, eating disorder.

The omnibus chi‐squared test of distinguishability was nonsignificant, Satorra‐Bentler *χ* ^2^(9) = 13.41, *p* = 0.34, which means that the parents were nondistinguishable by gender. Accordingly, all variances and covariances were constrained to be equal across genders in the APIM, that is, the mothers' actor effects and the fathers' actor effects were constrained to the same value, as well as the mothers' partner effects and the fathers' partner effects. Consequently, we henceforth refer to participants as Parent 1 and Parent 2, with no regard to their gender. The constrained model adequately represented the data, as reflected by good fit indices, *χ* ^2^(9) = 13.41, *p* = 0.34; CFI = 0.99; TLI = 0.98; RMSEA = 0.04, 90% CI [0.00, 0.12].

**FIGURE 1:**
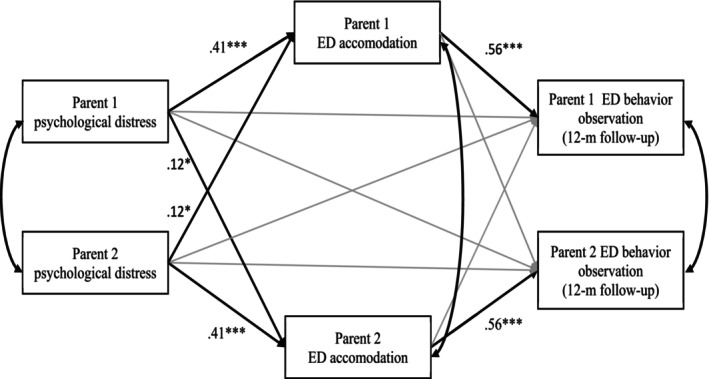
Actor–partner interdependence model. ED, eating disorder; *n* = 91 couples. This model was adjusted for the severity of the child's ED behaviors and symptoms at admission, as reported by the child's attending ED clinician using the EDS3 total score. The regression coefficients were standardized. Black lines represent significant paths, and gray lines represent tested nonsignificant paths. *p < 0.05; ***p < 0.001.

Results of the APIM are presented in Table 3, including all tested direct and indirect effects, while Figure 1 depicts all significant paths. For actor effects, the results showed no direct effect of parental psychological distress on their perception of their child's eating disordered behaviors at the 12‐month follow‐up, but bootstrapping analysis revealed a significant indirect effect of parental psychological distress on their perception of their child's eating disordered behaviors through ED accommodation, IE = 0.23, 95% bootstrap CI (0.15, 0.35). For partner effects, a significant indirect effect of Parent 1's psychological distress on Parent 2's observation of his/her child's eating disordered behaviors through Parent 2's ED accommodation was found, IE = 0.07, 95% bootstrap CI (0.02, 0.13). Altogether, the model explained approximately 33% of the variance in the child's eating disordered behaviors reported by parents at the 12‐month follow‐up.

**TABLE 3:** Actor–partner interdependence model.

	*β* (SE)	*p*	*β* (SE)	*p*
	Parent 1's ED accommodation		Parent 2's ED accommodation	
Parent 1's psychological distress	0.41 (0.06)	0.000	0.12 (0.06)	0.034
Parent 2's psychological distress	0.12 (0.06)	0.034	0.41 (0.06)	0.000
	Parent 1's ED behaviors observation		Parent 2's ED behaviors observation	
Parent 1's psychological distress	−0.11 (0.09)	0.192	−0.05 (0.07)	0.451
Parent 2's psychological distress	−0.05 (0.07)	0.451	−0.11 (0.09)	0.192
Parent 1's ED accommodation	0.56 (0.12)	0.000	−0.05 (0.10)	0.641
Parent 2's ED accommodation	−0.05 (0.10)	0.641	0.56 (0.12)	0.000

*Note:* The model was adjusted for the severity of the child's ED behaviors and symptoms at admission, as reported by the child's attending ED clinician using the EDS3 total score. Covariances were estimated in the model as reported in Figure 1.

Abbreviation: ED, eating disorder.

We examined several potential covariates (child age, diagnostic [typical vs. atypical AN], treatment setting at admission [outpatients vs. inpatients], duration of the ED and weight loss), but found that most did not correlate with the outcomes of interest in our model (i.e., the child's eating disordered behaviors reported by parents at the 12‐month follow‐up), with the exception of the treatment setting at admission. Adjustment for treatment setting at admission did not influence the strength or significance of the model (see Data S1).

## 4 Discussion

Using a dyadic model, the purpose of the present study was to provide a better understanding of the influence of both parents' emotional state and behaviors at the admission of their child to specialized eating disorder programs on their perception of their child's AN behavior 1 year later, in couples of parents who were still in a romantic, cohabiting relationship. First, it must be outlined that our analyses showed that parents were nondistinguishable by gender, suggesting a similar pattern of associations in mothers and in fathers. For actor effects, the results indicated that parents' psychological distress at the admission of their child to specialized eating disorder programs had no direct effect on their perception of their child's eating disordered behaviors 12 months later. However, an indirect effect was observed, that is, a higher psychological distress among the parents was associated with a greater risk of engaging in accommodating and enabling behaviors which, in turn, was associated with a heightened perception of eating disordered behaviors in their child 12 months later. The dyadic analysis also revealed a significant partner effect; when one parent experienced a higher psychological distress, the other parent reported to be more engaged in accommodating behaviors toward the child’s eating disorder symptoms, which in turn was associated with a heightened perception of the child's eating disordered behaviors by this second parent 12 months later.

These findings are consistent with the cognitive‐interpersonal maintenance model of Schmidt and Treasure (Janet et al. 2020; Schmidt and Treasure 2006; Treasure and Schmidt 2013), as it suggests that caregivers' psychological distress and accommodating and enabling behaviors may represent maintaining factors of AN, and that specific interventions targeting these factors could be highly relevant in the treatment of these patients. Good psychological health is required to be able to maintain a rigorous and consistent eating framework, especially in the context where their child, in the active phase of the illness, may show resistance to abandoning his/her eating disordered behaviors. Parents may seek to reduce their distress by reprioritizing caregiving aims to make life easier and avoid conflict with their child (Fox and Whittlesea 2017; Treasure and Schmidt 2013). They can then start to accommodate AN behaviors by adapting family routines to obey ED rules around food and eating, providing comfort and reassurance to their child surrounding eating, weight and shape, and removing consequences to the AN behaviors (Treasure et al. 2008). These accommodating behaviors can alleviate the distress in the short term but inadvertently reinforce symptoms by allowing the child to engage in AN behaviors, thus contributing to the maintenance of higher levels of eating disordered behaviors over time (Treasure and Schmidt 2013). Our findings can contribute to the understanding of the model, suggesting that the emotional state and behaviors of both mothers and fathers at the admission of their child to specialized eating disorder programs are associated with a heightened perception of the child's eating disordered behaviors by parents 1 year later. These results are highly relevant, considering that parents are usually the primary caregivers of the child suffering from AN, and they play a major role in the treatment of their child. In addition, by empirically measuring an integrative model that takes into consideration the interdependence between parents, we found that the parent's accommodating and enabling behaviors seem to be influenced not only by his/her own psychological distress but also, to some extent, by the distress of his/her partner. Parents may want to accommodate AN behaviors to alleviate their own stress and negative affect, but also to reduce the stress and negative affect of their partner, by minimizing potential conflicts with the child (Treasure and Schmidt 2013). Globally, these findings underscore the importance of adopting a dyadic perspective in exploring the emotional states of parents and their behaviors toward children with AN.

Our findings support those of previous studies that highlighted the relevance of addressing the psychological distress of parents of children and adolescents suffering from AN. Encouraging direct support for parents should begin in the early stages, from the detection of the AN, and be more easily accessible (Wilksch 2023). Interestingly, a meta‐analysis has suggested that caregivers' distress can be alleviated by a variety of psychoeducational interventions (Hibbs et al. 2015), and most of these interventions included a form of self‐directed help using materials that are widely available and could then be easily adopted by specialized eating disorder services. Providing parent‐only support sessions throughout the child's AN treatment has also been shown to reduce psychological distress (Wilksch 2023). Support groups for parents also seem to be of interest, allowing parents to share their story in a safe place and get support from other parents who are or have been in the same situation as them (Binford Hopf et al. 2013). In addition to psychological distress, our findings also suggest that parental accommodating behaviors could be associated with a heightened perception of the child's eating disordered behaviors 1 year later. Interestingly, additional analyses performed in the present study showed that the accommodation themes most strongly endorsed by parents were avoidance and modifying family routine (i.e., the extent to which caregivers change their typical activities and behaviors because of their child's disorder) and control of the family (i.e., the extent to which their loved one with ED controls the choice of food and cooking practice), followed by reassurance seeking (i.e., the extent to which families engage in repeated conversations focused on body image, eating, and negative thoughts and feelings). These parental behaviors could thus be hypothesized as relevant targets for the treatment of children and adolescents suffering from AN. Further studies are needed to test this hypothesis.

The mechanism of change in FBT is often attributed to the enhancement of parental self‐efficacy (Lock et al. 2015; Robinson et al. 2013). Parental self‐efficacy refers to a parent's belief in their ability to effectively manage and cope with the challenges posed by their child's ED. By empowering parents to take control and actively participate in their child's recovery, FBT aims to strengthen the family unit and create a supportive environment for the adolescent. Parental self‐efficacy has been previously linked with reductions in child's symptoms, depression and anxiety symptoms, and greater weight gain during treatment (Lock et al. 2015; Robinson et al. 2013). Parental distress can influence the sense of self‐efficacy among parents of children with ED by creating an emotional and psychological context that may compromise their perception of their ability to cope with the challenges associated with caring for their child (Coleman and Karraker 1998; Gondoli and Silverberg 1997). Emotional distress can lead to a decrease in self‐confidence and an altered perception of parental effectiveness, thereby impacting parents' ability to make informed decisions and implement effective strategies in managing their child's ED. Globally, our findings underscore the significance of attending to the well‐being of both parents.

We cannot overlook that participants in this study were predominantly White and well‐educated, a common limitation in ED research that may limit the generalizability of the findings to more diverse populations. Families from different racial/cultural and socioeconomic backgrounds may experience treatment settings in distinct ways. Research has shown that race/ethnicity and socioeconomic status can significantly influence access to care, treatment adherence, and outcomes, particularly in ED treatment (Lau et al. 2024; Moreno et al. 2023; Sonneville and Lipson 2018). Families of color and those with fewer financial resources may face additional barriers, including cultural differences, language disparities, and lack of access to culturally competent care. Furthermore, when treatment settings are predominantly staffed by White clinicians, there may be a mismatch in understanding the unique challenges faced by families from diverse backgrounds, which can impact the therapeutic relationship and the efficacy of family‐based strategies in the treatment of ED. Little is known about the influence of race/ethnicity and education on parental distress and accommodation behaviors in the treatment of pediatric ED. We believe that future research should aim to include more diverse populations to better understand these dynamics and improve the cultural sensitivity of family treatment approaches for ED.

### 4.1 Limitations

Although this study contributes to the understanding of the associations between parents' emotional state and behaviors at the admission of their child to specialized ED programs, and parents' observation of their child's eating disordered behaviors 1 year later using the APIM model, the most sophisticated statistical analysis technique for studying dyad relationships, some limitations should be outlined.

First, the final sample represents only a subset of families seen in the ED who agreed to participate, introducing the potential for selection bias. We cannot exclude the possibility that nonresponding parents differed in important ways from those who took part, which should be considered when interpreting the results and assessing their generalizability to the broader population of families affected by AN. Additionally, among the 123 couples eligible based on the inclusion criteria, 32 were excluded from the dyadic analyses because at least one parent did not complete any of the relevant questionnaires. As no sociodemographic or clinical data were available for these nonrespondents, it remains possible that they differed significantly from those who completed the study measures, further limiting the generalizability of our findings. It is important to note that the rate of refusal and missing data in our study is not unexpected, given the specific characteristics of our sample and the clinical context. Participants were recruited from hospital settings and included youth experiencing acute mental illness, along with their parents, who serve as their primary caregivers. The substantial burden placed on these parents likely contributed to the difficulties encountered in recruitment and data completion.

Second, only associations have been examined in our APIM model, which precludes the understanding of causality when examining the association between parental psychological distress at admission, parental accommodation at admission, and the parents' observation of the child's eating disordered behaviors 12 months later. The potential bidirectional relationship between parental distress and accommodation, where parental accommodation may also contribute to increased distress—either by causing tension between parents or by the accommodating parent experiencing distress from their own actions—was not fully explored. Addressing this possibility could deepen our understanding of the dynamics between parental distress and accommodation. It is crucial to consider this in future research, where the predictor variable could be measured before the mediator over time. This temporal separation is important because it helps establish the directionality of the relationship, reducing the risk of reverse causality and allowing for a clearer interpretation of how parental accommodation might influence subsequent distress, or vice versa.

Third, only a subsample of this cohort with data from mixed‐gender couples of parents who were still in a cohabiting relationship was included in these analyses, limiting the generalization of results. Since the present study aims to shed some light on a phenomenon that has not yet been investigated from a dyadic perspective, and that intact families represent two‐thirds of families in Canada (Statistics Canada 2016), we decided to initially focus on them. Further studies will be needed to verify whether these results apply to other types of families. In addition, children with AN in the present study were predominantly girls, and parents included in our analysis were mostly White and highly educated. Therefore, caution should be taken not to generalize these results to same‐gender couples, male patients, other racial groupings, and/or less educated parents. Finally, the high attrition rate for both mothers and fathers at the 12‐month follow‐up is a notable limitation.

### 4.2 Clinical Implications

Parental emotional state and their evaluation of their child's symptoms may be associated with their responses and engagement in treatment (Anastasiadou et al. 2014; Gondoli and Silverberg 1997; Zabala et al. 2009), suggesting avenues for clinical reflection. Since parents play a key role in supporting, reinforcing, and applying therapeutic interventions, our findings suggest that assessing and documenting parental emotional states, while also considering how these states interact with their interpretation of their child's symptoms, could be clinically useful. According to our results, distressed parents may engage in accommodating behaviors, sometimes at the expense of their confidence in setting boundaries. Feelings of distress and the accommodating behaviors that stem from them can be experienced as shameful, leading to guilt and reluctance to raise these challenges during treatment. Although our results do not allow us to establish causal directions between psychological distress and accommodating behaviors, it is also possible that such behaviors, particularly when persistent or unsupported, may contribute to increased parental distress, highlighting the potential for bidirectional influences. They suggest that clinicians may benefit from proactively addressing these behaviors, normalizing their presence, and providing parents with strategies to manage them constructively in ways that promote more compassionate self‐perception. Furthermore, our findings support the potential value of involving both parents in clinical discussions to better understand and address the emotional and behavioral dynamics within the caregiving context. Interventions that foster open communication, shared coping strategies, and a unified caregiving approach may be beneficial, though future research is needed to examine their effectiveness more conclusively. Taken together, the present findings suggest that helping both parents, as a caregiving dyad, to explore the links between their emotional states, their interpretations of symptoms, and their behavioral responses may contribute to a more stable and supportive family environment during ED treatment.

## 5 Conclusions

Overall, these findings indicate that both mothers and fathers show similar associations between their emotional state and behaviors when their child enters specialized eating disorder programs, and their observation of their child's eating disordered behaviors 1 year later, in intact, mixed‐gender, two‐parent families. Specifically, the results suggest that parents characterized by a higher psychological distress may be at greater risk of engaging in accommodating and enabling behaviors, which may, in turn, be associated with higher eating disordered behaviors observation over time. Perhaps most noteworthy, we observed interdependence between parents. Our findings propose that parents of a child with AN may accommodate AN behaviors to alleviate their own stress and negative affect, but also to reduce the stress and negative affect of the other parent.

Our findings highlight the need to consider not only mothers but also fathers in our research efforts. Past studies were mostly done with predominantly mother cohorts, and little is known about how fathers react to the AN diagnosis and how their reactions subsequently influence their child's eating behaviors. More importantly, the dyadic approach used in the present study highlighted the interdependence between parents, which should be considered in future research to gain a deeper understanding of their synergistic interactions. Additional studies could also consider the clinician assessments of ED symptoms in pediatric samples and explore their relationship with parental distress and accommodation, providing complementary insights. This could thereafter lead to more refined recommendations in the treatment of children and adolescents with AN.

Clinically, these findings suggest the importance of addressing both parents' psychological well‐being and their accommodating behaviors during the treatment of children and adolescents with AN and highlight the significance of considering the potential influence one parent may have on the other.

## Conflicts of Interest

The authors declare no conflicts of interest.

## Supporting information


**Data S1:** famp70070‐sup‐0001‐supinfo.docx.

## Data Availability

The data that support the findings of this study are available on request from the corresponding author. The data are not publicly available due to privacy or ethical restrictions.
