# Supplementary material for: Mother–Father Distress, Accommodation, and Child Eating Disorder Behaviors: A Dyadic Perspective
Source: Fam Process. 2025 Sep 4;64(3):e70070. doi: 10.1111/famp.70070 (PMC12411752; doi:10.1111/famp.70070)
Supplement: Supplementary file 1 — Data S1: famp70070‐sup‐0001‐supinfo.docx. [file FAMP-64-0-s001.docx]

**Supplemental Materials**

**Table S1**

*Actor-Partner Interdependence Model, Adjusted for Treatment Setting at Admission*

|  | Parent 1’s ED accommodation | | Parent 2’s ED accommodation | |
| --- | --- | --- | --- | --- |
|  | β *(SE)* | *p* | β *(SE)* | *p* |
| Parent 1’s psychological distress | 0.42(0.06) | .000 | 0.12(0.06) | .034 |
| Parent 2’s psychological distress | 0.12(0.06) | .034 | 0.42(0.06) | .000 |
|  | Parent 1’s ED behaviors observation | | Parent 2’s ED behaviors observation | |
|  | β *(SE)* | *p* | β *(SE)* | *p* |
| Parent 1’s psychological distress | -0.09(0.09) | .322 | -0.03(0.07) | .676 |
| Parent 2’s psychological distress | -0.03(0.07) | .676 | -0.09(0.09) | .322 |
| Parent 1’s ED accommodation | 0.54(0.13) | .000 | -0.07(0.10) | .485 |
| Parent 2’s ED accommodation | -0.07(0.10) | .485 | 0.54(0.13) | .000 |

*Note*. ED = eating disorder. As the model reported in the paper, this model was also adjusted for the severity of the child’s ED behaviors and symptoms at admission, as reported by the child’s attending ED clinician using the EDS3 total score.

**Figure S1**

*Actor-partner Interdependence Model, Adjusted for Treatment Setting at Admission*

.12*

.12*

.54***

*Note.* ED = eating disorder; n = 91 couples. As the model reported in the paper, this model was also adjusted for the severity of the child’s ED behaviors and symptoms at admission, as reported by the child’s attending ED clinician using the EDS3 total score. The regression coefficients were standardized. Black lines represent significant paths, and gray lines represent tested non-significant paths. Altogether, the model explained approximately 31% of the variance in the child’s eating disordered behaviors reported by parents at 12-month follow-up (R^2^ = 0.31). The constrained model adequately represented the data, as reflected by good fit indices: χ^2^ (9) = 17.44, *p* = 0.29; CFI = 0.98; TLI = 0.97; RMSEA = .04, 90% CI (.00 to .11).

* *p* < .05. *** *p* < .001.

Parent 1

ED accomodation

.54***

.42***

.42***

Parent 1

psychological distress

Parent 2

psychological distress

Parent 2 ED behavior observation

(12-m follow-up)

Parent 1 ED behavior observation

(12-m follow-up)

Parent 2

ED accomodation
